# Diagnostic Delay in Acromegaly Due to Overlapping Features With Polycystic Ovary Syndrome: A Case Report

**DOI:** 10.7759/cureus.92529

**Published:** 2025-09-17

**Authors:** Solomon O Siwoku, Ross-Michael Desvignes, Maria Silveira

**Affiliations:** 1 Medicine, University Hospitals Sussex NHS Foundation Trust, Worthing, GBR; 2 Endocrinology and Diabetes, University Hospitals Sussex NHS Foundation Trust, Worthing, GBR

**Keywords:** acromegaly, bitemporal hemianopia, diagnostic delay, igf-i sds, insulin-like growth factor-i standard deviation score, lanreotide, multidisciplinary care, non-secreting pituitary macroadenoma, polycystic ovary syndrome (pcos)

## Abstract

Acromegaly is a rare endocrine disorder marked by excessive secretion of growth hormone, typically as a result of a pituitary adenoma. This condition in female patients often presents with features that may overlap with polycystic ovary syndrome (PCOS), potentially leading to misdiagnosis or delayed diagnosis. This case study looks at a 33-year-old woman initially diagnosed with PCOS due to hirsutism, secondary amenorrhea, and features of polycystic ovaries on ultrasound. One year later, she experienced progressive visual disturbances and headaches, leading to the discovery of a large pituitary macroadenoma via MRI. Subsequent hormonal evaluation revealed elevated insulin-like growth factor 1 (IGF-1) and growth hormone levels, culminating in a diagnosis of acromegaly. The patient underwent successful endoscopic debulking of the adenoma with no residual disease detected on subsequent imaging. Post-operative management included Lanreotide therapy for persistently elevated IGF-1 levels. This case emphasises the importance of considering acromegaly in women with menstrual irregularities and hyperandrogenism, highlighting the need for comprehensive assessments to facilitate early diagnosis and address potential complications, such as vision loss and fertility issues.

## Introduction

Acromegaly is a rare endocrine disorder caused by the excessive secretion of growth hormone (GH), most commonly due to a pituitary adenoma. Its clinical features, such as menstrual irregularities, hirsutism, and insulin resistance, can be comparable with those of polycystic ovary syndrome (PCOS), resulting in potential misdiagnosis and delays in the timely identification of acromegaly in female patients. Sources have reported an incidence of PCOS ranging from 33 to 50% in women diagnosed with acromegaly [1,2], far exceeding the global incidence of 5.5 to 11.5% amongst reproductive age women [3]. Though not well understood, one explanation for this finding is that the excess growth hormone produced in acromegaly results in increased insulin resistance and consequent hyperinsulinemia [4]. This hyperinsulinemia may result in insulin acting directly at the ovaries, potentially increasing ovarian androgen production, which can cause hirsutism [5]. Elevated GH and IGF-1 levels themselves may also directly affect ovarian morphology [6]. This case illustrates the diagnostic challenges that arise when acromegaly co-occurs with PCOS, highlighting the need for thorough assessments and prompt evaluations by specialists.

## Case presentation

A 33-year-old woman was referred by her GP to the outpatient gynaecology team in July 2022 for evaluation of hirsutism and secondary amenorrhea, of one year's duration. Investigations at that time revealed normal serum total testosterone levels (1.23 nmol/L), low prolactin levels on two tests (75 mU/L and 97 mU/L), and low luteinizing hormone (LH) and follicle-stimulating hormone (FSH) levels at 0.7 IU/L and 2.6 IU/L, respectively. A pelvic ultrasound showed polycystic ovaries, leading to a diagnosis of polycystic ovary syndrome (PCOS). 

**Figure 1:**
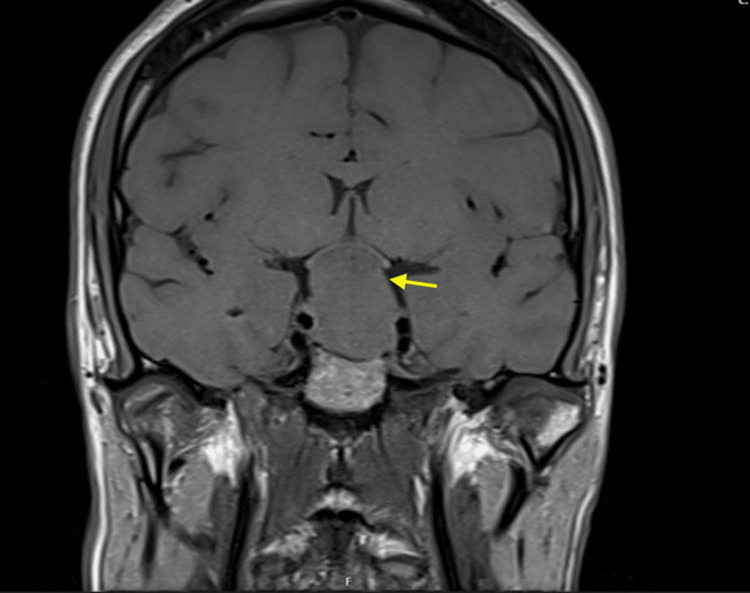
Coronal view of the preoperative pituitary MRI Arrow pointing towards a 5cm giant pituitary adenoma compressing the optic chiasm.

**Figure 2:**
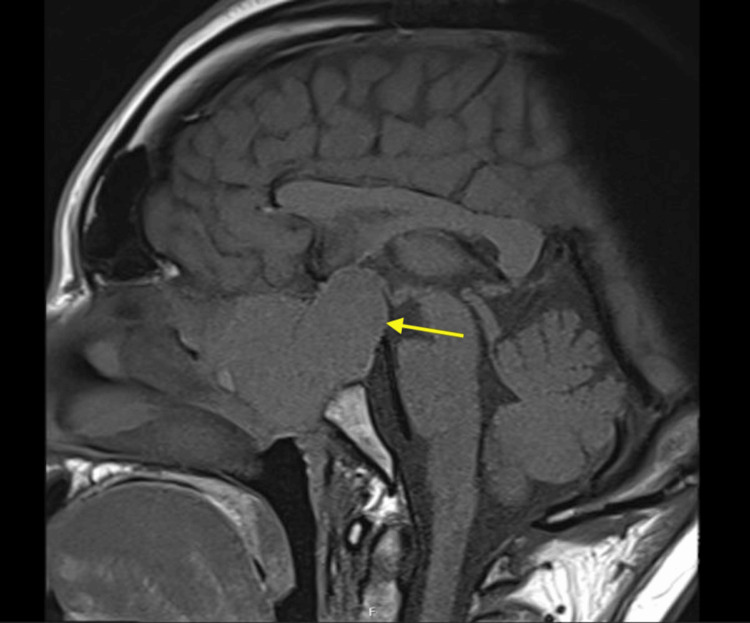
Sagittal view of the preoperative pituitary MRI Arrow pointing towards a giant pituitary adenoma eroding the planum sphenoidale, sella turcica, clinoid process and extending into the sphenoid sinus.

Approximately one year after her initial diagnosis, in July 2023, she presented to an optician due to progressive blurred vision and headaches. Goldman's visual fields demonstrated a significant loss of the left superior temporal field and right central field. She was urgently referred to the ophthalmology team, and a pituitary MRI scan was requested, which revealed a large pituitary macroadenoma compressing the optic chiasm, with extensive local invasion (Figures 1, 2). This led to a referral to the Pituitary Multi-Disciplinary Team Meeting and a pituitary screen. 

Anterior pituitary screen revealed elevated random growth hormone (GH) levels of 200 mcg/L and elevated insulin-like growth factor 1 (IGF-1) levels of 105.2 nmol/L. Thyroid-stimulating hormone (TSH) and adrenocorticotropic hormone (ACTH) were both within normal ranges, at 0.52 mU/L and 16 ng/L, respectively. Following this, she was diagnosed with acromegaly, and endoscopic debulking under the neurosurgeons was planned. Of note, during her maiden endocrine clinic review, an additional history of increasing shoe size in adulthood in the preceding two to three years was revealed. She was started on lanreotide 60 mg monthly preoperatively, to be discontinued postoperatively. 

**Figure 3:**
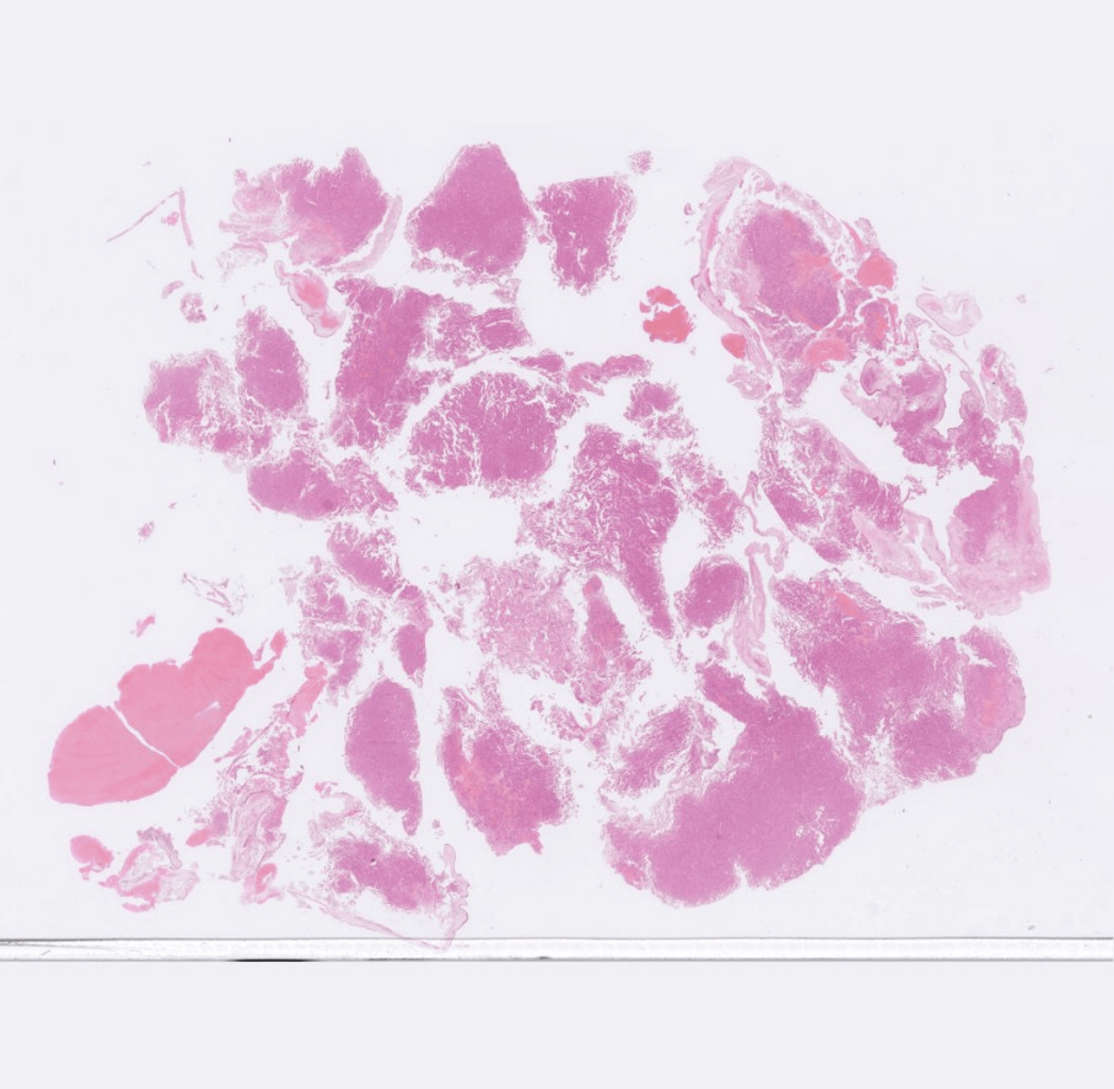
Haemotoxylin and Eosin-stained tissue sample Image showing neuroendocrine cells arranged in sheets, compact lobules, and pseudopapillae, occasionally with perivascular arrangement.

**Figure 4:**
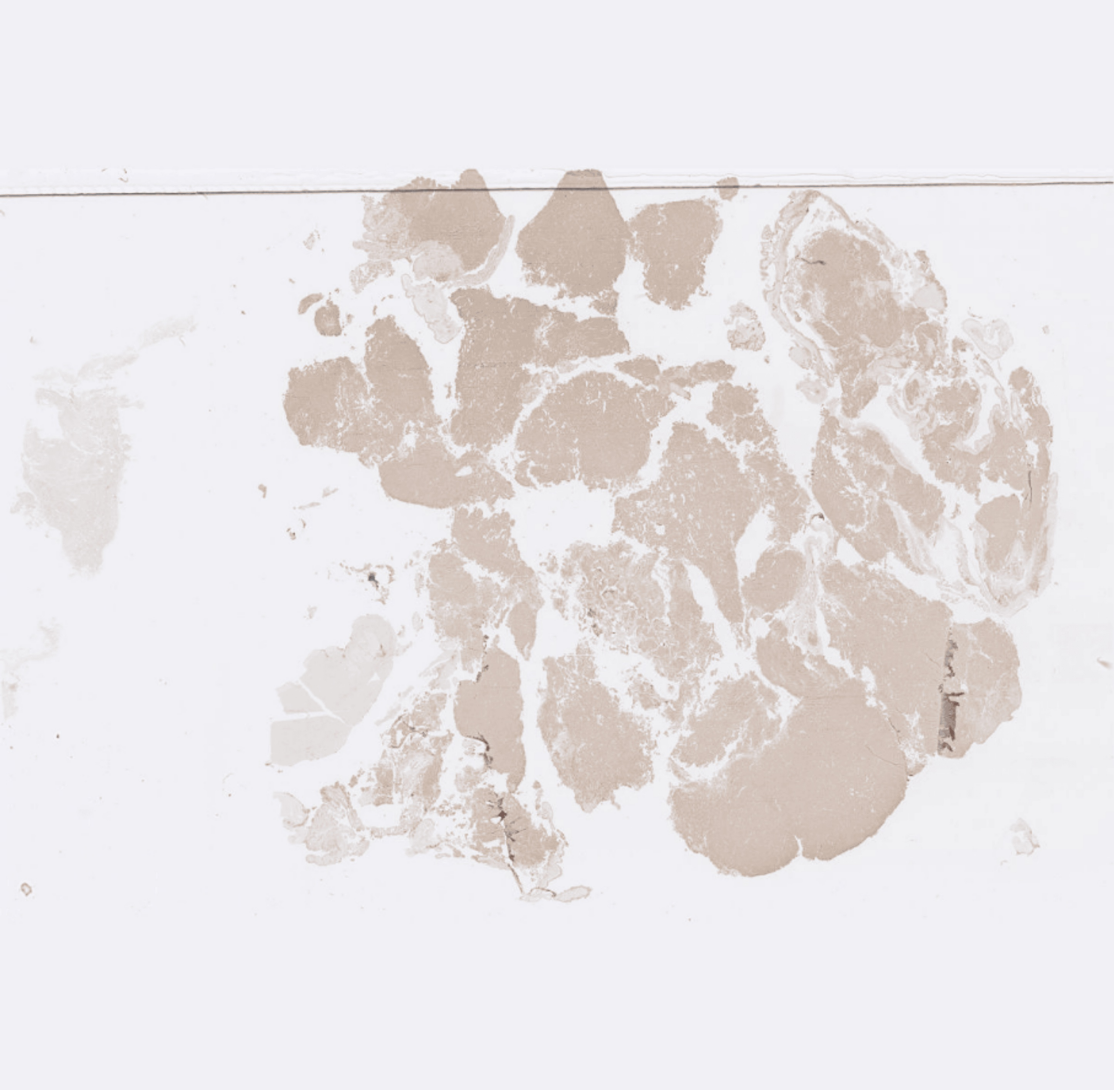
Growth hormone immunohistochemical staining Image showing positive growth hormone immunohistochemical staining.

In mid-October of 2023, she successfully underwent binasal endoscopic debulking of a giant pituitary macroadenoma. Histological results confirmed the presence of a pituitary neuroendocrine tumour (PitNET), characterised by the expression of steroidogenic factor 1 (SF-1) and pituitary transcription factor 1 (PiT-1) and diffuse growth hormone expression on immunohistochemistry in keeping with a diagnosis of acromegaly (Figures 3, 4). Postoperatively, she was placed on a standard dose of hydrocortisone to manage secondary adrenal insufficiency. Her thyroid function remained normal, and she showed no signs of vasopressin insufficiency. Following the surgery, her visual symptoms, as well as her impaired sense of smell and taste, improved significantly. The resolution of visual symptoms was confirmed on a repeat Goldman fields in June 2025, which demonstrated resolution of previous defects. In addition, her menstrual cycle also returned to normal. She was closely monitored for any signs of disease recurrence or complications, and her response to the treatment was regularly assessed with blood tests tracking IGF-1 levels. 

**Figure 5:**
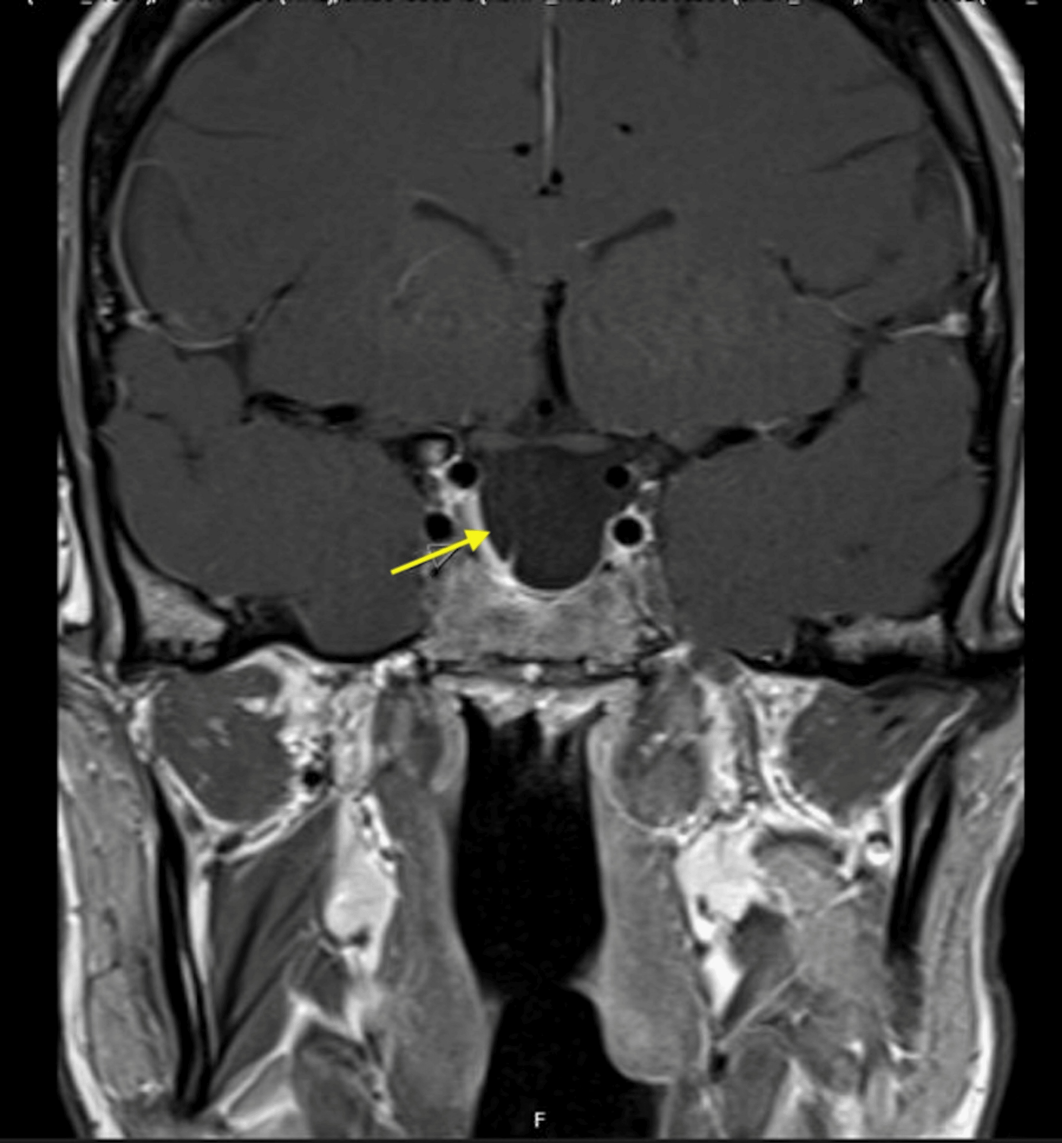
Coronal view of the postoperative pituitary MRI Arrow pointing towards a partial empty sella in a post-operative pituitary MRI following complete excision of a pituitary adenoma.

**Figure 6:**
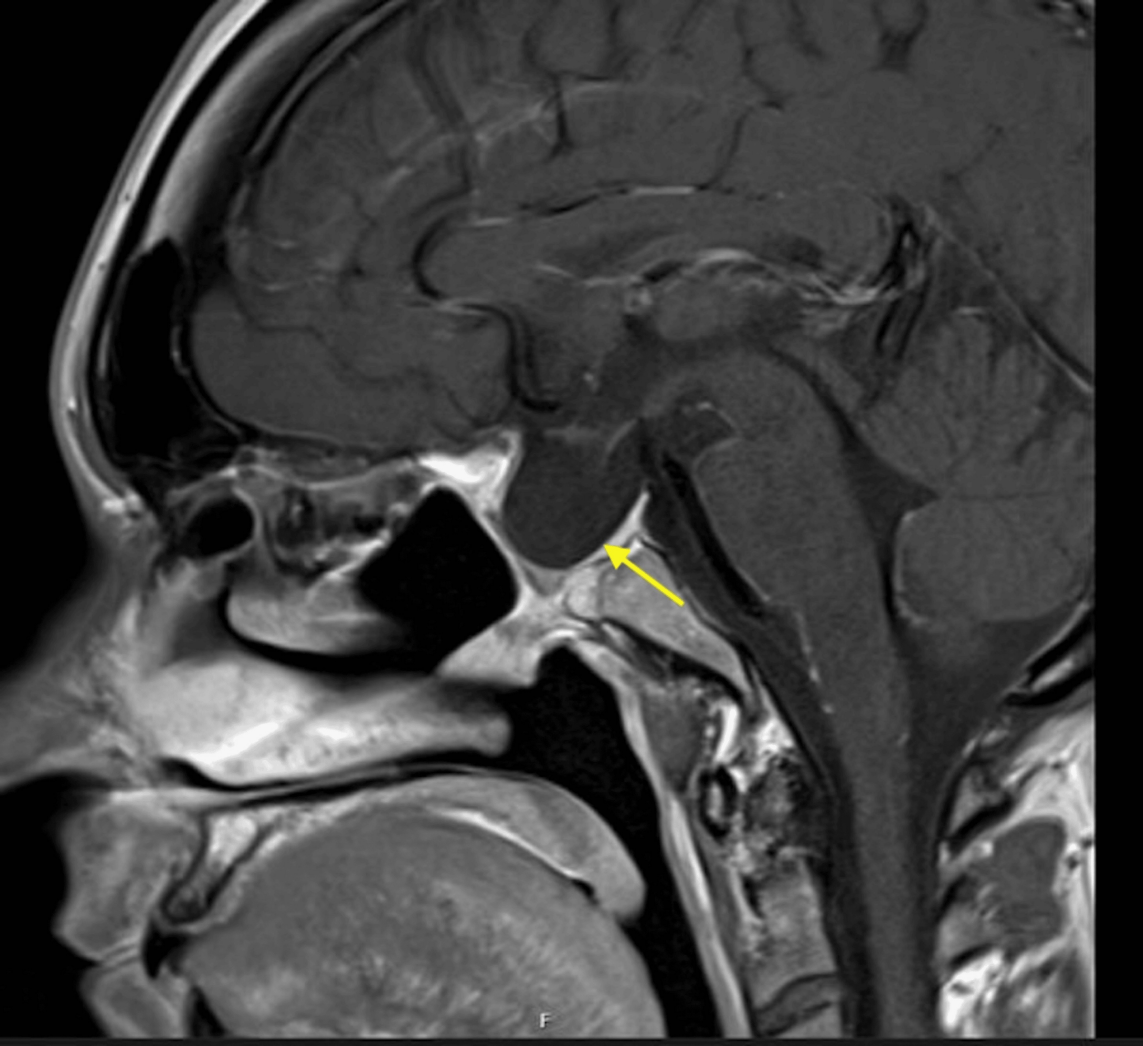
Sagittal view of the postoperative pituitary MRI Arrow pointing towards a partial empty sella after complete excision of a pituitary macroadenoma.

**Figure 7:**
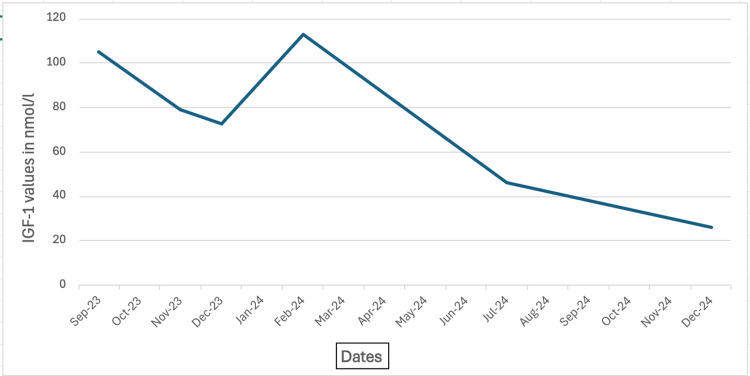
IGF-1 results trend Line graph showing a gradual reduction in IGF-1 levels over the course of 16 months. IGF-1 - insulin-like growth factor 1

Follow-up MRI scans showed no residual disease (Figures 5, 6); however, due to persistently elevated IGF-1 levels, she was restarted on lanreotide at a dose of 60 mg monthly. There is a plan for her to have methionine PET/CT imaging to investigate for residual disease and to inform future treatment options. Nine months into her Lanreotide therapy, her IGF-1 levels normalized (Table 1, Figure 7). 

**Table 1:** IGF-1 results timeline IGF-1 - insulin-like growth factor 1

Date	September 2023	November 2023	December 2023	February 2024	July 2024	December 2024	IGF-1 reference range
IGF-1 value in nmol/l	105.2	79.1	72.7	112.9	46.4	25.8	13.6 - 33.4 nmol/l

## Discussion

Polycystic ovarian syndrome (PCOS) according to the Rotterdam Consensus is defined by the presence of two of three of the following criteria: oligo‐anovulation(usually manifested as infrequent or no menstruation), clinical or biochemical hyperandrogenism and polycystic ovaries (≥ 12 follicles measuring 2‐9 mm in diameter and/or an ovarian volume >10 mL in at least one ovary) [7,8]. 

In contrast, acromegaly is a rare condition caused by excessive secretion of growth hormone (GH) and insulin-like growth factor 1 (IGF-1), often due to a pituitary adenoma (>99% cases) [9]. It usually manifests as acral enlargement (an increase in size of the hands and feet), changes in the face that are typical of the disease, and enlargement of internal organs (visceromegaly). Patients may also report nonspecific symptoms, including acne, menstrual irregularities, and hirsutism. In a retrospective study by Khiyami et al. (February 2023), about 60% of participants reported a history of irregular menstruation, and 30% reported hirsutism before they were diagnosed with acromegaly. 

On average, the time from the onset of symptoms to diagnosis ranges from 2.9 to 5.5 years [10,11]. This diagnostic delay is partly due to the subtle onset and nonspecific symptoms that resemble more commonly encountered conditions like PCOS. Another major contributor to this diagnostic delay is misdiagnosis. In a cross-sectional study done by Wang et al. (2021) involving 447 valid respondents, 58.8% of patients reported experiencing a misdiagnosis. The study also found that higher GH levels at diagnosis and the presence of endocrine-metabolic, musculoskeletal, and cardiovascular comorbidities were significantly associated with diagnostic delay (all p<0.05) [12]. Prompt recognition is therefore critical, as untreated acromegaly can lead to serious complications, including visual loss, infertility, cardiovascular and irreversible musculoskeletal changes, and even premature death [13].

Metabolic and reproductive disturbances, such as menstrual dysfunction and hyperandrogenism, can often be reversed in most patients after the surgical removal of the pituitary adenoma. A retrospective study by Khiyami et al. (2023) found that, following surgery, 54.5% of patients who met PCOS criteria experienced a return of menstruation and showed either clinical or biochemical evidence of resolved hyperandrogenism [1]. In our case, features such as increasing shoe size in adulthood, in addition to the low FSH and LH levels, were subtle clues towards acromegaly and pituitary dysfunction. Interestingly, the patient's initial prolactin levels were reported as low, with all subsequent tests returning within a normal range. Medication use did not explain this, and though likely a spurious result, it can also hint at pituitary dysfunction. Despite the initial diagnostic delay, the patient responded well to both surgical and medical treatments. 

In this case, the expression of steroidogenic factor-1 on histology may provide further explanation for the patient's presentation. This nuclear transcription factor plays an important role in the development of the reproductive system and regulates steroid biosynthesis [14]. Overexpression can lead to infertility, hyperandrogenism, and ovarian abnormalities [15]. SF-1 expression may be associated with gonadotrophic pituitary tumours and adrenal hyperplasia [16]; however, it is also commonly expressed in non-functioning pituitary adenomas [17], and the patient was never found to have cortisol excess or any adrenal pathology. 

Pit-1 is a pituitary-specific transcription factor involved in the generation, differentiation, and proliferation of three pituitary cell types: lactotrophs, thyrotrophs, and, significantly, somatotrophs, which produce growth hormone. [18]. It is overexpressed (2.5- to 5-fold) in prolactin and growth hormone-secreting tumors, but to an extent consistent with the predominant cellular type of these adenomas [18]. The expression of Pit-1 and SF-1 together has been termed as multi-lineage; however, these tumors exhibit a gene expression pattern and transcription factor activity profile very similar to other somatotroph tumors, with no resemblance to gonadotroph tumors apart from the expression of SF-1and certain SF-1-regulated genes such as LHB and GNHRH [19]. 

## Conclusions

In women presenting with menstrual irregularities and hyperandrogenism, it is imperative to consider alternative diagnoses to PCOS, including acromegaly, where the clinical picture and biochemical results do not align. A comprehensive evaluation should include inquiries regarding any atypical systemic features or neurological symptoms, as this information is crucial in identifying individuals who require screening for the disorder. Careful attention is warranted to prevent misdiagnoses and diagnostic delay. Standard laboratory tests such as follicle-stimulating hormone (FSH), luteinizing hormone (LH), and prolactin, which are employed in the assessment of polycystic ovary syndrome (PCOS), can provide valuable insights into possible pituitary dysfunction when interpreted correctly. Furthermore, insulin-like growth factor 1 (IGF-1) is a straightforward and effective screening tool that facilitates the differentiation of acromegaly from more prevalent conditions such as PCOS. 
